# COVID-19 in Switzerland and Liechtenstein: A Cross-Sectional Survey among Dentists’ Awareness, Protective Measures and Economic Effects

**DOI:** 10.3390/ijerph17239051

**Published:** 2020-12-04

**Authors:** Thomas Gerhard Wolf, Oliver Zeyer, Guglielmo Campus

**Affiliations:** 1 Department of Restorative, Preventive and Pediatric Dentistry, School of Dental Medicine, University of Bern, CH-3010 Bern, Switzerland; guglielmo.campus@zmk.unibe.ch; 2 Department of Periodontology and Operative Dentistry, University Medical Center of the Johannes Gutenberg-University Mainz, 55131 Mainz, Germany; 3 Swiss Dental Association SSO, CH-3000 Bern, Switzerland; oliver.zeyer@sso.ch; 4 Department of Medicine, Surgery and Experimental Sciences, School of Dentistry, University of Sassari, I-07100 Sassari, Italy; 5 Faculty of Dentistry, Sechenov First Moscow State Medical University, Moscow 119991, Russia

**Keywords:** awareness, COVID-19, dentist, economic effect, global pandemic, infection control, Liechtenstein, protective measures, Switzerland

## Abstract

The aim of this observational cross-sectional study was to gain information on the awareness; protective measures and economic effects of dentists in Switzerland during the global COVID-19 pandemic. All dentist were members of the Swiss Dental Association SSO from all over Switzerland—including all Swiss cantons and Liechtenstein—and received a previously calibrated questionnaire as an ad hoc online version. The questionnaire was divided into four parts: personal data; precautionary measures; awareness; perception. In total, 1324 questionnaires were analyzed; the response rate was 30.59% (*n*^total^ = 4328). Participants stated in less than 2% common symptoms/signs of COVID-19; of which only fatigue was statistically significant (*p* < 0.01). A small number of dentists reported a positive test (0.91%; *n* = 12) or having one or more symptoms (2.65%; *n* = 35) of COVID-19 during the pandemic; whereas only 6.71% (*n* = 87) of the participants reported having treated SARS-CoV-2 infected patients. High prevalence areas were only medium-large and large Swiss cantons (*p* < 0.01). Face filter (FFP2/FFP3) masks were used by about half of the dentists, while disposable visor was rarely used. The majority of dentists had to reduce the dental practice activity to a minimum of 0–10% (*n* = 923; 69.98%) due to the lockdown. This economic impact forced 1.4% (*n* = 18) to close their practice permanently or by the end of 2020 due to the economic situation. These results can be helpful to better prepare dental practices for future outbreaks of infection (e.g., prophylactic storage of additional protective measures), define the best strategy and organize the dental workforce. Political decision-makers should consider drastic economic effects when deciding on drastic measures such as “lockdown”, which can lead to practice closures and unemployment of dental staff after only a few weeks. This should be taken into account, especially with regard to possible financial assistance to severely impaired dental practices to maintain a high level of dental care.

## 1. Introduction

The epidemic of COVID-19 was reported due to cases of pneumonia with unknown cause to the World Health Organization (WHO) on 31 December in 2019 [1] with the number of confirmed cases being 44 by 3 January in Wuhan City (Hubei Province, China). On 12 March, this was finally characterized by the WHO as a controllable pandemic in which the affected member states should continue to focus on containment rather than alleviation [2]. COVID-19 rates reported from different countries are not easily comparable, which may lead to biased conclusions. The positive rate in a population is a good metric of the spread of the virus. The new beta coronavirus SARS-CoV-2 (Severe Acute Respiratory Syndrome COronaVirus type 2) was identified as the trigger for COVID-19 in early 2020. The Swiss canton of Ticino in the south of Switzerland, bordering on northern Italy and the Lombardy region, not far from Milan (Italy), increasingly found SARS-CoV-2 positive individuals in early February. The increase was so rapid that, on 28 February, the Swiss Federal Council assessed the situation as a “special situation” under the Epidemics Act and adopted the Ordinance on Measures to Combat Coronavirus (COVID-19). As a result, major events with more than 1000 participants were banned. A campaign by the Swiss Federal Office of Public Health was launched on 1 March with hygiene recommendations for protection against SARS-CoV-2 infection [3]. The Swiss Dental Association SSO developed a protection concept for dental practices during the period of validity of the emergency legislation passed by the Federal Council on 16 March [4]. Furthermore, also on 16 March, the Swiss Federal Council defined the “extraordinary situation” due to the highest danger level with measures to protect the population in accordance with the Epidemics Act. As a result, all shops, restaurants, bars, as well as entertainment and leisure facilities, with the exception of grocery stores and health care facilities, were closed until 19 April 2020 (lockdown) [3]. Many dental practices only provided emergency treatment or even had to close down completely for some time, both due to possible COVID-19 infections of employees/dentists or the precarious financial situation of missing patient flows. However, no valid data are available yet. Due to the massive restrictions on public life imposed by this ordinance, all non-essential shops and services were closed with immediate effect. The “extraordinary situation” was extended once again until 26 April. According to the COVID-19 Ordinance, practices and facilities of health professionals must have a protection concept that is appropriate to the situation and operation [3]. Due to the so-called “Smart Restart” initiated by the Swiss Dental Association SSO, the individual implementation of the protective measures has made it possible to reopen dental practices for patient care since 27 April 2020, largely without restrictions [5]. Most of the emergency measures have been lifted since May 11, but major events will continue to be postponed until the end of August. Following a decision on 1 July, the Swiss Federal Council decided to make masks compulsory on all public transport, which has been in force since 6 July 2020 [3]. Despite the “controllable” global pandemic designated by the WHO in March, 41,771,932 confirmed cases of COVID-19 (in accordance with the applied case definitions and testing strategies in the affected countries), including 1,138,780 deaths, have been reported as of 23 October 2020 [6]. A scientific consensus on the COVID-19 pandemic warns against a second wave in Europe and strongly recommends clear communication on the risks of COVID-19 and effective control strategies [7].

The authors searched PubMed and its specific hub, Embase and Scopus servers up to 18 May 2020, for epidemiological studies using the terms “Dentists” or “Dental personnel” and “SARS-CoV-2” without date or language restrictions. Few or small studies focused on specific population subgroups were found. Large population-based studies are needed to estimate the infectivity rate among dentists, to collect symptoms/signs presumably related to the COVID-19 and to investigate the adopted preventive measures and personal protective equipment (PPE). The University of Bern designed and carried out a global survey to evaluate the impact of the COVID-19 outbreak among dentists working in different countries [8,9]. In Switzerland, the survey was accomplished in collaboration with the Swiss Dental Association SSO; therefore, the aim of this paper was to analyze, evaluate and describe the information about dentists’ awareness, protective measures and economic effects during the global COVID-19 pandemic in Switzerland and the Principality of Liechtenstein. The collected data should help to define the best strategy and to organize the dental workforce in both countries.

## 2. Materials and Methods

### 2.1. Development and Calibration of the Questionnaire

The global questionnaire was developed on the basis of a previous pilot study performed in Italy [8] at the beginning of the outbreak of the COVID-19 pandemic in the northern Italian hotspot, the region around Milan, Lombardy. In brief, the items related to the health situation, risk, was derived from the questionnaire developed for SARS risk [9] following the Stehr–Green scale. The questionnaire was structured into four domains. The first contains personal data (such as age, sex, residential area and work status), the second contains health status (such as symptoms/indications related to COVID-19 disease) [10], the third contains work status and personal protective equipment (PPE) adopted after the outbreak of the infection, and the fourth contains knowledge and self-perceived risk of infection. Based on these basic items, a preliminary questionnaire, translated into German and French, was prepared and developed for Switzerland and presented to a small group of dentists (*n* = 12). The intraclass correlation coefficient (ICC) for the repetition of the test and the intra-rater reliability of each item were calculated. An ICC value of 0.80 or higher was considered satisfactory. Items with an ICC value below 0.80 were discussed by the authors and modified and adjusted according to the preliminary results.

### 2.2. Online Survey

An anonymous online survey [11] was prepared. On 2 July, all dentists *n* = 4328 who were in the database of the Swiss Dental Association SSO in Switzerland and Liechtenstein—i.e., 58.77% of a total of 7364 dentists with granted licenses in the Register of Medical Professions in Switzerland (as of 31 December 2019) [12] and the Principality of Liechtenstein (*n* = 58; as of 2017) [13]—received an e-mail requesting their consent to participate in the questionnaire in accordance with the applicable data protection laws. All participants received a personal survey code by e-mail. After entering the code, the participant was taken to the online survey input interface, where they could enter the information directly on the computer. The personal survey code was only valid once. In addition, all information was completely coded and then anonymized. All participants were asked to declare that they had read the privacy policy and that they voluntarily agreed to the data collection and processing. Questionnaires that were not completely filled out or completed were not included in the data collection and therefore no data were collected. If they answered no, the questionnaire was automatically closed and no data were collected. The questionnaire was sent only once, a reminder was not sent. The participants were asked to respond by 6 July. Nevertheless, the questionnaire was unlocked until 15 July and then stopped. All procedures were in accordance with the ethical standards of the local research committee and with the 1964 Helsinki declaration and its later amendments, or with comparable ethical standards. Swiss law/Federal Act on Research involving Human Beings (Human Research Act) [14] does not require ethics committee approval to collect and analyze anonymous data. Informed consent was obtained from all study participants by accessing the online survey.

### 2.3. Data Analysis

The answers to the questionnaire were inserted into Excel^TM^ 2019 (Microsoft Corp., Redmond, WA, USA) for Macintosh (Apple Inc., Cupertino, CA, USA). The data were cleaned and then transferred to STATA16^TM^ (Stata Corp., College Station, TX, USA) for statistical analysis. The 26 Swiss cantons and the Principality of Liechtenstein were grouped according to the COVID-19 prevalence as follows [8,15]: Areas of a low prevalence of COVID-19 lower than 0.30% were grouped together (AG, AI, AR, LU, FL, NW, OW, SG, SH, SO, SZ, TG, TI, ZG). Regions with a prevalence between 0.33% and 0.62% were grouped together as middle prevalence (BL, BS, FR, GL, GL, GR, JU, NE, UR, VS, ZH). Regions with a high prevalence above 0.62% were grouped as high (GE, TI, VD). Absolute and relative frequencies were calculated for each item. The difference in ratio and proportion was evaluated using the χ^2^ test or the exact Fisher test if a cell had a value of less than five. The symptoms most frequently reported in the literature (fever, cough, fatigue) [10] were used to compare areas with different prevalence of COVID-19 [16]. A *p*-value of less than 0.05 was considered statistically significant. The Swiss cantons and the Principality of Liechtenstein were divided into four groups derived from the agglomeration size class of the Federal Statistical Office of the Swiss Statistics of Population and Households according to the Federal Population Census [17]: small with less than 100,000 inhabitants (AI, AR, GL, JU, FL, NW, OW, SH, UR), medium-small with 100,000 to 300,000 inhabitants (BL, BS, GR, NE, SO, SZ, TG, ZG), medium-large (FR, LU, TI, VS) with 300,000 to 500,000 inhabitants and large with more than 500,000 inhabitants (AG, BE, GE, SG, VD, ZH).

## 3. Results

A total of 4328 emails with personal coding were sent, of which 1324 completed and closed the questionnaire (response rate 30.59%). Of these 879 (66.39%) were male and 445 (33.61%) female. No statistically significant differences were observed among the different age groups (*p* = 0.39). It was divided into three groups according to prevalence areas (Swiss cantons and the Principality of Liechtenstein) of COVID-19 infection: low, medium, and high. Of these, 609 (46.00%) were in low, 521 (39.35%) in medium and 194 (14.65%) in areas with high infection rates or high prevalence (data not in table).

**Figure 1:**
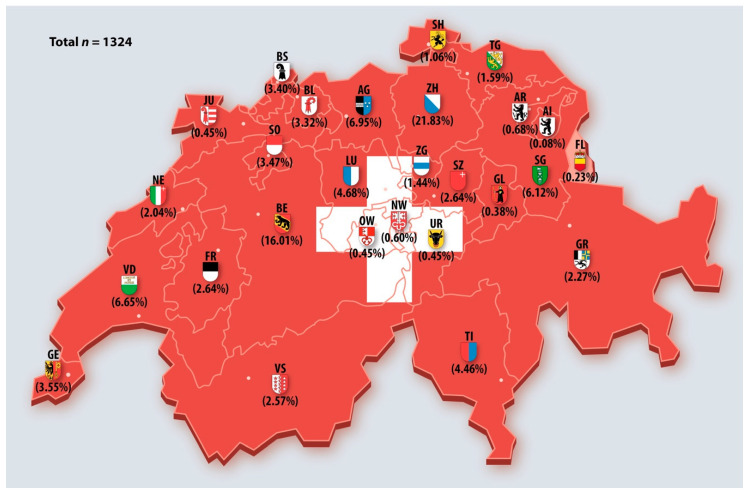
Map of Switzerland and the Principality of Liechtenstein with the percentage of participating dentists in the current study (*n* = 1324).

Figure 1 shows the distribution of participating dentists in Switzerland and the Principality of Liechtenstein divided by Swiss cantons (only Switzerland).

Among the symptoms/signs (Table 1), cough and fatigue were the most common (1.21% and 1.51%, respectively), while fever, breathing difficulties and nasal congestion were stated less frequently (0.83%, 0.68% and 0.53%, respectively). The information on COVID-19 specific symptoms/signs was very low, at >2%. While there was little difference between the prevalence of COVID-19 between Swiss cantons and the Principality of Liechtenstein, only the data for fatigue were statistically significant (*p* < 0.01). From the five common symptoms from the literature (fever, cough, fatigue, breathing difficulties and nasal congestion), the only statistically significant symptom reported from dentists in Switzerland and Liechtenstein was fatigue (*p* < 0.01) compared with low to middle and high prevalence areas (Table 1).

Almost half of the dentists in Switzerland and the Principality of Liechtenstein have continued their practice with increased observance of the prescribed hygiene regime and the additionally recommended measures (*n* = 653; 49.66%). Significantly fewer dentists stopped their practice for a period of more than two weeks (*n* = 280; 21.29%) or continued their work but had limited themselves to dealing with emergencies that cannot be postponed (*n* = 251; 19.09). While only 1.98% (*n* = 26) continued their work without changing the type and time of treatment, 3.19% (*n* = 42) had to close their practice due to economic hardship. While 57 dentists (4.33%) had to close their practice for two weeks due to their own COVID-19 disease, 0.46% (*n* = 6) had to close their practice for two weeks due to a positively tested practice employee (*p* < 0.01) (Table 2). The majority of dentists had to reduce the practice activity to a minimum of 0–10% (*n* = 923; 69.98%) (Table 2). In total, 14.63% of the dentists (*n* = 193) have limited their practice activity to 11–30% and 3.79% (*n* = 50) to 31.60% (Table 3). Only 11.60% (*n* = 153) had little to no reduction. The differences between the groups were statistically significant (*p* < 0.01).

With regard to the different size of the site where dentists perform their practice activities on patients, there are statistically significant differences (*p* < 0.01) between the individual prevalence areas, as shown in Table 4.

The percentage of workload during the lockdown among Swiss cantons and the Principality of Liechtenstein was not statistically significant associated with COVID-19 prevalence (data not in table). Table 5 reports the standard protective measures for symptom-free patients since the beginning of the COVID-19 pandemic and stratified by COVID-19 prevalence area and area by size. Interestingly, the distribution regarding the use of face filter (FFP2/FFP3) masks among all prevalence areas was quite balanced, although the use of the sight in low prevalent areas is much less frequent. However, for both parameters there was no statistically significant difference between areas of different COVID-19 prevalence.

More than one third of the participants (*n* = 450) stated that they had attended online training on COVID-19 (e.g., Skype or Zoom Meeting), 62.89% (*n* = 832) did not attend such training, 3.10% did not give any information (*n* = 41). By other means, 94.03% (*n* = 1244) acquired knowledge about COVID-19, 3.40% (*n* = 45) did not and 2.57% (*n* = 34) did not give any information. From the professional associations and professional societies 84.13% (*n* = 1113) feel sufficiently informed about COVID-19, 13.45% (*n* = 178) denied this and 2.42% (*n* = 32) did not give any information.

Only 6.71% (*n* = 87; *n*^total^ = 1296) of dentists treated patients with SARS-CoV-2 (data not in table). In total, 1.4% (*n* = 18, *n*^total^ = 1290) of the participating dentists stated that, due to the economic situation caused by the COVID-19 pandemic, they are either permanently closing their practice or will be forced to close their practice in the near future (by the end of the year 31 December 2020) and are looking for a buyer to sell their practice. Of these, six were in a low, eight in a middle and four in a high COVID-19 prevalence area (χ^2^_(2)_ = 1.43 Pr = 0.49) (data not in table). Table 6 displays the ordinal logistic regression for COVID-19 prevalence area, the area by size, work during lockdown, state of health, use of face filter (FFP2/FFP3) masks and disposable visor.

In high prevalence areas, the parameter’s area by size, work during lockdown, state of health and the use of face filter (FFP2/FFP3) were statistically significant (*p* < 0.01).

## 4. Discussion

### 4.1. Awareness of COVID-19

The present survey was carried out almost three months after the “smart restart” of all dental practice activity in Switzerland and Liechtenstein following the lockdown due to the global COVID-19 pandemic. The southern part of Switzerland borders on the Italian region of Lombardy, mainly in the Swiss canton of Ticino, which had the highest number of SARS-CoV-2 infections and deaths in northern Italy at the beginning of the spread of the COVID-19 pandemic in early spring 2020. The sample of dentists to whom the questionnaire was sent by e-mail was more than half of the dentists in Switzerland and Liechtenstein, according to data from the Swiss Federal Office of Public Health (FOPH) and the Office for Statistics of the Principality of Liechtenstein [12,13]. The response rate was quite low, even though the sample is quite representative for Switzerland and Liechtenstein at just under 18%. The data of this study were conducted as part of a large-scale global survey and collected with participating countries on all continents of the world [9]. These data for Switzerland were collected in cooperation with the University of Bern (Switzerland) and the Swiss Dental Association SSO. To the best of the authors’ knowledge, this is the only study to date that collects information on awareness, protective measures and economic effects in Switzerland and Liechtenstein during the global COVID-19 pandemic [9]. Although it is not surprising that 94% of the participants have acquired information about COVID-19 in different ways, as the media coverage has been dominated by information about SARS-CoV-2 and the related disease COVID-19 for months, it is interesting to note that more than one third have acquired the information digitally via online training, which is an indication that many dental training events have been switched from presence to online as a result of the various country regulations of the pandemic. It remains to be seen how this development might change in the future. Still, no data on the health conditions of dentists with regard to COVID-19 are available to date. The percentage of people who tested positive or had COVID-19 (0.91%) in the study is similar to the areas of populations with high prevalence of COVID-19 and is in agreement with a study in the North of Italy [8]. This data could be due to a possibly higher participation rate in the survey of persons infected with the virus or with claimed symptoms/signs. However, they were reported with a relatively very low percentage of dentists (3.56%). It must be taken into consideration that the symptoms/signs were also caused by other diseases, such as seasonal influenza or other infections of viruses or even bacteria. Surprisingly, the symptoms/signs were generally around 5% and the differences between areas with different prevalence were marginal.

### 4.2. Protective Measures

The use of protective measures, such as face filter (FFP2/FFP3) masks, was confirmed by about half of the dentists, as previously reported in the literature [8]. There is a large discrepancy in the use of disposable visors/headset in the present survey, which was about 10–12% and 64% in northern Italy [8]. This may be due to the fact that, by declaring the area as a high prevalence area early on, dentists were very aware of the situation in Lombardy (Italy) regarding the possible risks and effects of the virus infection. Another reason could have been the different training with protective materials as well as the possible availability of such materials. In this context it should be considered that there was a worldwide shortage of protective materials due to the high daily need in many countries. The multinomial regression analysis underlined that the use of N95/FFP2 was significantly associated in the different Switzerland areas, grouped by COVID-19 prevalence. However, in Switzerland, there is no additional local/national recommendation for the use of visors or headsets in the dental practice. While dentists in the present report provided similar information on protective materials in all COVID-19 prevalence areas, in Norway, a small proportion of dental staff in high prevalence areas have been equipped with additional infection prevention measures compared to those in low and medium prevalence areas [18].

### 4.3. Economic Effects on Dental Practice

The majority of dentists had to reduce the practice activity to a minimum of 0–10%. This is in agreement with a study in Poland with a total of 71.2% of the participating dentists that decided to entirely suspend their dental practice [19]. The main reasons for this were unpreparedness of the dental sector, shortage of personal protective equipment, subjective perception of the risk of COVID-19 infection, and a general feeling of fear and insecurity in the situation at that time [19]. Unfortunately, while the present survey did not examine the reasons for the reduction in practice activity in more detail, Poland saw a significant decrease in the number of patients treated weekly compared to before the pandemic started in March [19]. Although not examined in detail in the current study, the decrease in patient numbers during the lockdown has also been reported by dentists in Switzerland and Liechtenstein. The reasons for this and the economic impact should be further investigated. The significant patient decline is also reported in a Norwegian study; twice as many patients were treated by telephone compared to clinical care [18]. This fact is also reported from China [20], where numerous public dental hospitals stopped the elective treatment of patients and only offered emergency treatment. Almost 90% of the hospitals reported that they offered dental consultations online, 70% of which were free of charge. Long-term effects on the dental care of patients are feared by the authors in China [20]. Impacts on practice structures are possible, although currently not foreseeable and not further investigated in the current study. However, the effects on smaller practice structures such as individual practices may be different from those of larger ones, such as dental centers [21]. The uncertain situation may also have a decisive influence on the young dental profession and may also change expectations of the profession and possibly discourage the establishment of a practice in the near future, thus changing the landscape of dental professions as well as oral health care in Switzerland and Liechtenstein [22,23]. The effects of the lack of patient flows that would have economic effects could primarily be the changeover to short-time work, reduction in working hours or even the dismissal of salaried dentists and employees. This could also lead to problems, such as unemployment in the long term, although this is not yet foreseeable. The backlog of treatments resulting from a lack of treatment could also lead to the loss of the patient base due to restricted opening hours, and the resulting financial shortfalls could ultimately lead to insolvency and the closure of the practice. While 1.4% of the study participants will be forced to permanently close by the end of 2020 at the latest, due to the economic situation, this situation will have to be evaluated again in the autumn/winter in order to identify effects early and, if necessary, to take countermeasures from the political side in order to continue to successfully maintain oral health care.

### 4.4. Limitations

A limitation of the study is the short time span of data collection, which developed due to the constantly changing situation in the different cantons of Switzerland and the Principality of Liechtenstein over the summer during the COVID-19 pandemic. On the one hand, it is necessary to take into account the perceptions of dentists that changed significantly during the outbreak and the lockdown over the summer and that the COVID-19 regulations and constantly changing different measures placed great demands on the staff of dental practices. Although the number of participating dentists is representative for Switzerland and Liechtenstein, it is possible that our study comprised mainly dentists who experienced little impact or who did not want to disclose their personal sensitive data regarding the COVID 19 pandemic. Through the experience of previous studies, the authors [9] limited the number of questions that they felt were most relevant in order to obtain the best possible results and experiences that could help dentists and policy makers to improve the situation in a possible second wave or to make better decisions based on the newly obtained knowledge. Most importantly, it is worth mentioning that the study was conducted when the situation in Switzerland and Liechtenstein had eased in July 2020. The findings on SARS-CoV-2 and COVID-19 have not yet been fully researched and understood, and a transfer of knowledge among dentists is still necessary. This is therefore only a snapshot and not a general statement.

## 5. Conclusions

Participants reported common symptoms/signs of COVID-19 in less than 2% of cases. A relatively small proportion of dentists reported a positive COVID-19 test or one or more symptoms of COVID-19 during the pandemic. Only a small number of dentists treat patients infected with SARS-CoV-2. High prevalence areas were only medium-sized and large Swiss cantons. Face filters (FFP2/FFP3) masks were used by about half of the dentists, while disposable visors were rarely used. The majority of the dentists had to reduce the activity in the dental office to a minimum of 0–10% due to the closure. The economic impact forced 1.4% of the study participants to close their practices permanently or by the end of 2020 due to the economic situation.

The results can be helpful to better prepare dental practices for future outbreaks of infection. This includes the awareness and knowledge of infectious diseases as well as the necessity of use and the prophylactic storage of possible additional protective measures and hygiene articles for dental treatment. The economic effects are also essential for a functioning oral health system. The reasons for this and possible assistance for dentists should be further investigated. The data collected in this study may, therefore, play an important role in the increasing awareness among professionals and policy makers of the impact of the COVID-19 pandemic on the dental profession in order to define the best strategy and organize the dental workforce.

## Figures and Tables

**Table 1:** Prevalence of symptoms/signs related to the COVID-19 in the different Swiss cantons and the Principality of Liechtenstein sorted after COVID-19 prevalence level. Percentages were calculated per column.

Symptoms/Signs	Low Prevalence Area		Middle Prevalence Area		High Prevalence Area		Total	
	*OF*	*%*	*OF*	*%*	*OF*	*%*	*n*	*%*
**Fever**	3	0.23	4	0.30	4	0.30	11	0.83
Cramer’s V = 0.06 Fisher’s exact = 0.12								
**Cough**	8	0.60	3	0.23	5	0.38	16	1.21
Cramer’s V = 0.06 Fisher’s exact = 0.08								
**Fatigue**	4	0.30	8	0.60	8	0.60	20	1.51
Cramer’s V = 0.09 Fisher’s exact > 0.01								
**Breathing difficulties**	2	0.15	4	0.30	3	0.23	9	0.68
Cramer’s V = 0.05 Fisher’s exact = 0.14								
**Nasal congestion**	3	0.23	1	0.08	3	0.23	7	0.53
Cramer’s V = 0.06 Fisher’s exact = 0.09								

**Table 2:** Working conditions from the beginning of the COVID 19 pandemic until the beginning of July 2020 (survey time of questionnaire) stratified by Swiss cantons (including the Principality of Liechtenstein) with different prevalence of the disease. Percentages were calculated per column.

COVID 19	Normal Working		Working Additionally Measures		Only Emergencies		Stop Working for More than 2 Weeks		Practice Close Due to Economic Hardship		Practice Close (Positively Employee)		Practice Close (Positively Owner)	
	*OF*	*%*	*OF*	*%*	*OF*	*%*	*OF*	*%*	*OF*	*%*	*OF*	*%*	*OF*	*%*
**Low prevalence**	11	1.81	328	54.04	105	17.30	126	20.76	19	3.13	1	0.16	17	2.80
**Middle prevalence**	9	1.75	250	48.64	98	19.07	119	23.15	15	2.92	3	0.58	20	3.89
**High prevalence**	6	3.09	75	38.66	48	24.74	35	18.04	8	4.12	2	1.03	20	10.31
***p-value***	*χ^2^_(2)_ = 66.21 < 0.01*		*χ^2^_(2)_ = 48.33 < 0.01*		*χ^2^_(2)_ = 18.73 <0.01*		*χ^2^_(2)_ = 7.97 0.02*		*χ^2^_(2)_ = 36.66 < 0.01*		*0.09*		*χ^2^_(2)_ = 61.37 < 0.01*	

**Table 3:** Dentist working load (express in percentage) during the lockdown compared to the situation immediately before the workload, which corresponds to 100% work activity.

COVID 19 Prevalence	Workload < 10%		Workload < 30%		Workload 60%		Normal Workload (100%)	
	*OF*	*%*	*OF*	*%*	*OF*	*%*	*OF*	*%*
**Low prevalence**	428	46.37	106	54.92	23	46.00	49	32.03
**Middle prevalence**	379	41.06	71	36.79	18	36.00	9	18.00
**High prevalence**	116	12.57	16	8.29	51	33.33	53	34.64
***p-value***	*χ^2^_(2)_ = 297.82 < 0.01*		*χ^2^_(2)_ = 496.71 < 0.01*		*χ^2^_(2)_ = 37.84 < 0.01*		*χ^2^_(2)_ = 8.11 0.02*	

**Table 4:** Swiss cantons and the Principality of Liechtenstein divided by size (small, medium-small, medium-large and large) stratified by different prevalence of the coronavirus disease. Percentages were calculated per column.

COVID 19	Small		Medium-Small		Medium-Large		Large	
	*OF*	*%*	*OF*	*%*	*OF*	*%*	*OF*	*%*
**Low prevalence**	41	6.73	121	19.87	62	10.18	385	63.22
**Middle prevalence**	17	3.26	146	28.02	69	13.24	289	55.47
**High prevalence**	--	--	--	--	59	30.41	135	69.59
***p-value***	*χ^2^_(2)_ = 23.12 < 0.01*		*χ^2^_(2)_ = 1.75 0.19*		*χ^2^_(2)_ = 16.04 < 0.01*		*χ^2^_(2)_ = 4.82 0.09*	

**Table 5:** Standard protective measures for symptom-free patients since the beginning of the COVID-19 pandemic and stratified by COVID-19 prevalence area and area by size. Percentages were calculated per column.

		Use of Face Filter (FFP2/FFP3)				Disposable Visor			
COVID 19	Area	No		Yes		No		Yes	
		*OF*	*%*	*OF*	*%*	*OF*	*%*	*OF*	*%*
**Low prevalence**	**Small**	34	58.62	24	41.38	39	95.12	2	4.88
	**Medium-small**	143	53.56	124	46.44	108	89.26	13	10.74
	**Medium-large**	88	46.32	102	53.68	54	87.10	8	12.90
	**Large**	363	44.87	446	55.13	330	85.71	55	14.29
	***p-value***	*χ^2^_(3)_ = 12.69 p < 0.01*				*χ^2^_(3)_ = 3.52 p = 0.32*			
**Middle Prevalence**	**Small**	25	60.98	16	39.02	12	70.59	5	29.41
	**Medium-small**	77	63.64	44	36.36	113	77.40	33	22.60
	**Medium-large**	29	46.77	33	53.23	59	85.51	10	14.49
	**Large**	185	48.05	200	51.95	249	86.16	40	13.84
	***p-value***	*χ^2^_(3)_ = 10.97 p = 0.01*				*χ^2^_(3)_ = 54.73 p < 0.01*			
**High Prevalence**	**Small**	9	52.94	8	47.06	--	--	--	--
	**Medium-small**	66	45.21	80	54.79	--	--	--	--
	**Medium-large**	38	55.07	31	44.93	47	79.66	12	20.34
	**Large**	143	49.48	146	50.52	114	84.44	21	15.56
	***p-value***	*χ^2^_(3)_ = 1.99 p = 0.57*				*χ^2^_(1)_ = 0.37 p = 0.54*			

**Table 6:** Multilevel ordinal logistic regression about the COVID-19 prevalence area, the area by size, work during lockdown, state of health, use of face filter (FFP2/FFP3) masks and disposable visor. The table includes the fixed-effect portion of our model, the estimated cut-points and the estimated variance components.

COVID-19 Prevalence Area	Variables	OR (SE)	*p*-Value	95% CI
Low Prevalence	*Base Outcome*			
**Middle Prevalence**	*Area by size*	0.90 (0.06)	0.08	0.79–1.01
	*Work during lockdown*	1.02 (0.02)	0.45	0.97–1.07
	*Presence of symptoms *	1.37 (0.40)	0.27	0.78–2.42
	*Use of face filter (FFP2/FFP3)*	1.11 (0.13)	0.403	0.87–1.41
	*Disposable visor*	1.32 (0.23)	0.78	0.69–1.65
**High Prevalence**	*Area by size*	1.80 (0.22)	<0.01	1.41–2.29
	*Work during lockdown*	1.17 (0.03)	<0.01	1.11–1.23
	*Presence of symptoms*	2.91 (0.88)	<0.01	1.61–5.26
	*Use of face filter (FFP2/FFP3)*	2.55 (0.48)	<0.01	1.77–3.68
	*Disposable visor*	1.15 (0.28)	0.54	0.72–1.85

## Abbreviations

COVID-19	Coronavirus Disease
SARS-CoV-2	Severe Acute Respiratory Syndrome Coronavirus 2
OF	Observed frequency
AG	Aargau
AI	Appenzell Innerhoden
AR	Appenzell Ausserrhoden
BE	Bern
BL	Basel-Landschaft
BS	Basel-Stadt
FL	Fürstentum (Principality of) Liechtenstein
FR	Fribourg
GE	Genève
GL	Glarus
GR	Graubünden
JU	Jura
LU	Luzern
NE	Neuchâtel
NW	Nidwalden
OW	Obwalden
SG	St. Gallen
SH	Schaffhausen
SO	Solothurn
SZ	Schwyz
TG	Thurgau
TI	Ticino
UR	Uri
VD	Vaud
VS	Valais
ZG	Zug
ZH	Zürich
